# Miscellaneous Items

**Published:** 1886-08

**Authors:** 


### MISCELLANEOUS ITEMS.

### *SOME MEDICAL ANECDOTES.*

[From the Parisian Journal *D'Hygiene.*]

**A physician** having a sick horse, committed him to the care of a farrier, who having cured the animal, his owner enquired of him, "My friend, how much do I owe you?" "Nothing," replied the farrier; "we never take money from those of the same profession."

**A patient** weary with long suffering, upon remarking the many and various medicines ranged about his bedside, regarded himself much the same as a condemned soldier in the midst of his armed executioners, and addressing himself to one of the more potent among them, after the manner of culprits about to undergo the death sentence, said, "Monsieur, I select you to blindfold me."

**A miserly old physician**, brusque and but little sought after, employed a little country boy to make himself useful, and answer calls in his absence. Returning one evening in very bad humor, on account of some disappointment, he questioned the lad, who being still without his dinner, answered with some show of bitterness. "How youngster, mind you, sir, tell me promptly who has been here in my absence," in turn demanded the physician.

"Well, then," answered the young starveling, "since you will have me say it, a priest has called to tell you that your patient is dead; an apothecary to say that your prescriptions are worthless; an old woman who wishes you to the devil, because you have poisoned her; a bailiff who came for money; but neither bread, nor wine, nor food of any kind has come, and I am nearly famished."

**An octogenarian physician** rejoiced in unalterable good health. Upon this his friends frequently complimented him. "Doctor," enquired they, "how do you manage to keep in such continued good health?" "I am free to tell you gentlemen," replied the doctor, "and at the same time to exhort you to profit by my example. I live simply, upon the income of my practice, without taking any of the remedies which I ordered for my patients."

**The first Emperor, Maximilian**, being sick, called in a number of physicians, more with an object of diversion, than the expectation of being benefited by their prescriptions. To each one of them in turn, he put forth the following word "*quot?*" They hesitated, confused, evidently having no conception of his majesty's purpose or meaning.

At length, a shrewd, old practitioner, conceiving that by the word "*quot*," the prince required of him to say how many persons he had hurried out of the world by his treatment, grasped a full hand of his beard, and answered "*tot*" thus signifying that the number equalled the individual hairs of his beard. The Emperor was so well pleased with the wit and sincerity of this reply, that the author of it was at once selected as his physician."

---

**Last Fall** the windows of a vacant house in Danbury, Conn., were broken by stones thrown at them. No one could be seen throwing the stones, and the windows were boarded up. The other day the boards were removed, and at once the stone throwing began again. The *News* says that the stones used are small, round pebbles. They are thrown with such velocity and precision that often two or three go through the same hole in a pane of glass. The stones are thrown while people stand talking, and they cannot see them pass through the air. It is presumed that they are fired from a gun operated by compressed air. Those who have stood and watched the windows and suddenly heard the breaking of the glass, without seeing the missile that did the mischief, are becoming quite superstitious over the matter.

**"Rocking."**—The ordinary use of the above term, is apt to recall scenes ineffaceably impressed upon our childish memories, but the rugged coast of Maine has had a tendency to give it a new and no less grateful signification. The opinion of the New York *World* upon the propriety of these latter-day indulgences has been asked by a young lady correspondent about to bring herself within the circle of their fascinations, at Mount Desert. According to her explanation "rocking" consists in two persons of opposite sexes selecting a secluded spot on the cliffs or rocks, and there indulging in conversation. She also adds that the charms of "rocking" are much enhanced by the raising of an umbrella. In response the *World* proceeds to remark. "We must confess to our inability to successfully cope with so puzzling a question, and we frankly admit that any objections we might raise would avail little to prevent or even interfere with the amusement. The books on etiquette are silent on the subject, and of two well-informed women whose opinion was requested, one said: "Rocking is perfectly harmless and is charming," and the other replied: "It is horrid and unrefined." It is only proper to add that the first lady interrogated is a belle of one winter and the second no longer counts her summers. In view of these contradictory opinions and of the absence of any defined rules on the subject of "rocking," we should say that if taken in small doses and in modertion, due care being exercised as to the choice of the second party to the amusement, our fair correspondent may indulge in "rocking" at Mount Desert with a clear conscience."

